# RBM15 facilitates laryngeal squamous cell carcinoma progression by regulating TMBIM6 stability through IGF2BP3 dependent

**DOI:** 10.1186/s13046-021-01871-4

**Published:** 2021-02-26

**Authors:** Xin Wang, Linli Tian, Yushan Li, Jingting Wang, Bingrui Yan, Like Yang, Qiuying Li, Rui Zhao, Ming Liu, Peng Wang, Yanan Sun

**Affiliations:** Department of Otorhinolaryngology, Head and Neck Surgery, The Second Affiliated Hospital, Harbin Medical University, Harbin, 150086 China

**Keywords:** Laryngeal squamous cell cancer (LSCC), N6-methyladenosine (m6A), RNA binding motif protein 15 (RBM15), TMBIM6, IGF2BP3

## Abstract

**Background:**

Laryngeal cancer has the highest mortality rate among head and neck tumours. RNA N6-methyladenosine (m6A) is the most plentiful and variable in mammalian mRNA. Yet, the m6A regulatory mechanism underlying the carcinogenesis or progression of LSCC remains poorly understood.

**Methods:**

The m6A RNA methylation quantification kit was used to detect tissue methylation levels. m6A microarray analysis, mRNA transcriptomic sequencing (mRNA-seq), and proteomics were used to determine RBM15, TMBIM6, and IGF2BP3. Immunohistochemical (IHC), quantitative real-time PCR (qRT-PCR) and Western blot were used to investigate RBM15, TMBIM6, and IGF2BP3 expression in tissue samples and cell lines. The biological effects of RBM15 were detected both in vitro and in vivo. The combination relationship between RBM15/IGF2BP3 and TMBIM6 was verified by RNA immunoprecipitation (RIP) assay, Methylated RNA immunoprecipitation sequencing (MeRIP-seq), RNase Mazf, and luciferase report assay. RNase Mazf was used to determine the methylation site on TMBIM6 mRNA. Hoechst staining assay was used to confirm the apoptotic changes. The actinomycin D verified TMBIM6 stability.

**Results:**

The global mRNA m6A methylation level significantly increased in LSCC patients. RBM15, as a “writer” of methyltransferase, was significantly increased in LSCC and was associated with unfavorable prognosis. The knockdown of RBM15 reduced the proliferation, invasion, migration, and apoptosis of LSCC both in vitro and in vivo. The results were reversed after overexpressing RBM15. Mechanically, TMBIM6 acted as a downstream target of RBM15-mediated m6A modification. Furthermore, RBM15-mediated m6A modification of TMBIM6 mRNA enhanced TMBIM6 stability through IGF2BP3-dependent.

**Conclusion:**

Our results revealed the essential roles of RBM15 and IGF2BP3 in m6A methylation modification in LSCC, thus identifying a novel RNA regulatory mechanism.

**Supplementary Information:**

The online version contains supplementary material available at 10.1186/s13046-021-01871-4.

## Background

Head and neck carcinomas are the sixth most common cancers by incidence among all types of malignant tumors, while the laryngeal cancer is the second most common cancer by incidence in the head and neck region. In 2017, approximately 13,360 new LSCC patients were diagnosed in the United States [1, 2], while in China, this number amounted to approximately 26,400 new LSCC patients in 2015. Although a variety of treatment methods have improved, the 5-year survival rate of LSCC patients has not significantly improved over recent decades [3, 4]. Therefore, it is urgent to find effective molecular markers and reveal the mechanisms underlying LSCC tumorigenesis or development so as to explore more effective strategies for early diagnosis, prognostic evaluation, and treatment.

N^6^-methyladenosine (m^6^A) is the most abundant RNA modification, which was the first revealed in mRNA and has also been recently found in precursor mRNAs, circRNAs, and lncRNAs [5–7]. m6A can be catalyzed by various enzymes, including methyltransferases, demethylases, and effector proteins. Furthermore, m6A modification can control RNA fate by affecting post-transcriptional regulation, such as RNA stability, splicing, and translation efficiency, which influences multiple biological processes, including cell differentiation, tissue development, as well as spermatogenesis, and RNA–protein interactions [8, 9]. Recent researches have demonstrated that m6A RNA methylation has an essential role in various human diseases such as hypertension [10], cardiac hypertrophy [11], viral infection [12], diabetes [13] and cancers [14]. For instance, Chen et al found that WTAP’s participation in m6A methylation has a vital role in the occurrence of hepatocellular carcinoma [15]. Furthermore, Wang et al reported that high METTL3 expression promotes tumor angiogenesis and glycolysis in gastric cancer [16]. Bai et al determined that YTHDF1 exerts a crucial oncogenic role in CRC by promoting Wnt/β-catenin pathway [17]. However, the RNA m6A expression patterns and their relevant mechanisms in LSCC remain largely unknown.

In this study, the overexpression of m6A methyltransferase RBM15 in LSCC tissues was first authenticated. The RBM15 level was associated with the progression of LSCC and the prognosis of LSCC patients. We further revealed that RBM15 exerts an oncogenic role in LSCC both in vitro and in vivo. From the mechanism, RBM15 can regulate the m6A level of TMBIM6 mRNA, which depends on the m6A reader IGF2BP3. Taken together, these results indicated that the RBM15/IGF2BP3/TMBIM6 axis might be a novel and promising therapeutic target for LSCC.

## Materials and methods

### Tissue samples

All 164 pairs of LSCC and adjacent non-tumour tissues were collected from the Department of Otorhinolaryngology, the Second Affiliated Hospital of Harbin Medical University. The selection criteria included patients diagnosed with laryngeal cancer before surgery and those who have never received any treatment from 2013 to 2016. The expression levels of m6A were performed in 34 patients, a total of 8 matched samples of LSCC tissues, and the corresponding adjacent non-tumor tissues were tested by microarray and proteomics detection. Moreover, we additionally gathered 34 pairs of LSCC tissues to determine the expression of RBM15, TMBIM6, and IGF2BP3 by qRT-PCR. LSCC tissues from 122 patients were used for IHC assay.

The Harbin Medical University Ethics Committee has endorsed this study.

### Cell culture and transfection

Human LSCC cells (AMC-HN-8 cells, TU-212 cells, and TU-177 cells) and NHBEC (normal human bronchial epithelial cell) cells were maintained in Dulbecco’s modified Eagle’s medium (DMEM) supplemented with 10% fetal bovine serum in a humidified incubator at 37 °C with 5% CO_2_. The cells were cultured in 24-well plates (5 × 10^4^ cells/well) overnight. RBM15, TMBIM6, and IGF2BP3 knockdown and overexpression viruses and their respective control vectors were provided by Genechem (Shanghai, China). Transfection protocol followed the transfection reagent instructions.

### m6A RNA methylation quantification

The m6A quantification was carried out utilizing the Abcam m6A RNA Methylation Quantification Kit (Abcam ab185912). The relevant solutions were added to each well according to the manufacturer’s instructions. The solution was mixed by gentle tilting from side to side, after which m6A RNA capture was performed. It was ensured that any residual wash buffer in the wells was thoroughly removed at each wash step. Signals were detected at the end when the color of the positive control wells changed to medium blue. A 100 μL Stop Solution was then added to each well to stop the enzyme reaction. After adding the Stop Solution, the color of the compound solution changed to yellow. The absorbance was read at 450 nm on the microplate reader.

### qRT-PCR

Total RNA from LSCC tissues and cell lines was extracted using Trizol reagent (Invitrogen, Carlsbad, CA, USA) according to the manufacturer’s instructions. The process of qRT-PCR was as previously reported [18]. Data were calculated using the 2^-ΔΔCt^ method. Sequences of the primers are listed in Additional file 1: Table S1.

### Microarray analysis

Human m6A epitranscriptomic microarray and mRNA microarray analysis were from Arraystar Arraystar Company (Rockville, MD, USA). Briefly, the total RNAs were immunoprecipitated with anti-N6-methyadenosine (m6A) antibody. The elution from the immunoprecipitation magnetic beads was called “IP”. The recovered supernatant was called “Sup”, and Labels “IP” and “Sup” RNA were used for Cy5 and Cy3, respectively. After merging, it was hybridized to Arraystar Human m6A Epitranscriptomic Microarray (8 × 60 K, Arraystar). Finally, an Agilent scanner G2505C was used to scan the array.

### Proteomics

The protein was extracted from the sample tissue. A 20 μg of protein was taken from each sample, which was mixed with 6 X loading buffer, boiled in a water bath for 5 min. Samples were then analyzed using a 12% SDS-PAGE electrophoresis (250 V, 40 min) and FASP enzymolysis. A 100 μg peptide was taken for each sample and was labeled according to the Thermo Company’s TMT labeling kit instructions. Data analysis was performed after mass spectral analysis and identification. An appropriate protein sequence database was selected, which is the foundation and critical step for the qualitative analysis of protein in mass spectrometry data.

### MeRIP-qPCR

A 1–3 μg total RNA and m6A spike-in control mixture was added to 300 μL 1IP buffer (50 mM Tris-HCl, pH 7.4, 150 mM NaCl, 0.1% NP40, 40 U/μL RNase Inhibitor) containing 2 μg anti-m6A rabbit polyclonal antibody (Synaptic Systems). A 20 L Dynabeads™ M-280 Sheep Anti-Rabbit IgG suspension per sample was blocked with freshly prepared 0.5% BSA at 4 °C for 2 h, washed three times with 300 μL 1IP buffer, and re-suspended in the total RNA-antibody mixture prepared above. The RNA binding to the m6A-antibody beads was carried out with head-over-tail rotation at 4 °C for 2 h. The enriched RNA was eluted with 200 μL Elution buffer at 50 °C for 1 h, after which the co-precipitated RNA was isolated and qRT-PCR detection was performed on the RNA as the previously described. Sequences of the primers are listed in Additional file 1: Table S1.

### Western blot

After the total protein was extracted, the protein concentration was measured with the BCA protein assay kit (Beyotime Biotechnology, China). Protein samples were electrophoresed, transferred to PVDF membranes, and blocked with 5% nonfat milk at RT for 1 h. Then the PVDF membrane was incubated with the primary antibody solution anti-RBM15 antibody (ab244374; Abcam, Shanghai, China, 1:1000), anti-TMBIM6 antibody (ab18852; Abcam, Shanghai, China, 1:1000) and anti-IGF2BP3 antibody (ab177477; Abcam, Shanghai, China, 1:1000) overnight at 4 °C. Monoclonal anti-rabbit IgG (RS0002; Immunoway, Plano, USA, 1:5000) or monoclonal anti-mouse IgG (3420; Abcam, Shanghai, China, 1:5000) were incubated with the PVDF membrane for a secondary step 1 h at room temperature. Finally, chemiluminescence (ECL) detection reagents (Beyotime Biotechnology, China) were prepared under dark conditions to detect blots.

### Luciferase reporter assay

AMC-HN-8 and TU-212 cells in 24-well plates were transfected with a luciferase reporter and indicated expression constructs. The assays were carried out as previously reported [18].

### Immunohistochemistry

The slice number was marked and placed on the grill for 30 min. The paraffin slides were deparaffinized and rehydrated, and then the antigen was retrieved in a pressure cooker. The protein was blocked with 10% goat serum, and the slides were incubated with anti-RBM15 primary antibody (10587–1-AP; Proteintech, Wuhan, China, 1:500) at 4 °C overnight. The expression evaluation was as previously described [19].

### CCK-8 assay

LSCC cells were cultured overnight on 96-well plate (100 μL per well contains 2000 cells). The culture medium and CCK-8 solution were used as a blank control group. On the next day, 10 μL CCK-8 solution was added to each well. The absorbance at 450 nm was measured at 0, 24, 48 and 72 h.

### Wound healing assay

A totoal of 5 × 10^5^ cells (per well) were seeded into a 6-well culture plate. After 48 h of incubation, the cells grew to about 90% confluence, and the middle of every well was scraped with a 200 μl sterile pipette tip. The plate was then washed three times with PBS to remove cell debris, an cultured in fresh DMEM containing 2% FBS. The wound was imaged at two-time points after 0 h and 20 h. Image-Pro Plus 6.0 was used to assess the gap distance quantitatively.

### Transwell assay

For cell invasion/migration analysis, LSCC cells were starved for 24 h. The bottom of the transwell filter with a pore size of 8 μm was coated with a 1:8 dilution of Matrigel (ignoring this step in cell migration assay). The single-cell suspension (1 × 10^5^; 200 ul) diluted in serum-free medium was added to the upper chamber, and a medium (600 ul) containing 15% FBS was added to the lower chamber. The cells were allowed to migrate for 24 h at 37 °C. The cells were fixed in 3.7% formaldehyde for 15 min, washed with PBS, and stained with crystal violet for 10 min, after which the upper chamber non-invasive/migrating cells were wiped. The number of cells in three replicate experiments was evaluated to quantify cell invasion or migration.

### In vivo experiment

Balb/c male nude mice, 5–6 weeks old, weighing 20–25 g, were obtained from Vital River Laboratories (Beijing, China). All the animals were housed in an environment with a temperature of 22 ± 1 °C, relative humidity of 50 ± 1%, and a light/dark cycle of 12/12 h. All animal studies (including the mice euthanasia procedure) were done in compliance with the regulations and guidelines of Harbin Medical University institutional animal care and conducted according to the AAALAC and the IACUC guidelines.

For tumor growth studies, whether in vivo RBM15 knockdown/overexpression experiments or in vivo rescue experiments, each group included six mice. Each mouse was injected with 100 μl of lentivirus-transfected tumor cells. The simplified volume of the spheroid (length × width ^2^ × 0.5) was used to determine the tumor volume. Six weeks after inoculation, xenografts were excised and evaluated for volume.

### TUNEL assay

After being deparaffinized twice with dimethylbenzene, alcohol gradient hydration was performed. The reagents from the TUNEL kit (Contains 5 μL of equilibrium solution and 45 μL of TdT enzyme) were added to all slides at 37 °C for 1 h (50 μL/slide). The anti-digoxin antibody was dropped on all the slides, and then placed in a wet box, and infiltrated at 37 °C for 20 min. DAB developer was added to the slide to react for 20 min. Hematoxylin was slightly counterstained, differentiated in a hydrochloric acid alcohol solution for 2 s, and rinsed with distilled water for 10 min. The tissues were then dehydrated and transparently treated. Finally, the apoptosis of the two groups was observed under the microscope.

### Transmission electron microscopy

The tumor was put in a refrigerator at 4 °C, fixed with glutaraldehyde for 24 h; the pH of the solution was adjusted to 7.3 with 0.1 mmol/L dimethyl arsenate buffer. The tissue was then thoroughly washed, and 1% tetraoxide Osmium was fixed in draught cupboard for 2 h at room temperature. After progressive dehydration, the tissues were embedded with the SPI-Pon812 embedding agent. The specimen was heated on an alcohol burner to detach it from the slide and was cut to a thickness of about 70 nm under the microtome. The tissue sections were stained with double electron staining of uranium acetate and lead citrate and rinsed three times, after which they were air-dried at room temperature. The ultrastructure of the transplanted tumor cells was observed under a transmission electron microscope.

### RNase Mazf

Cellular RNAs were digested at the unmethylated ACA site using bacterial single-stranded RNase MazF. Sites with m6A methylation remain uncleaved. Based on the ability of MazF to discriminate between 5′-ACA-3′ and 5′-(m6A)CA-3′, we determined the m6A methylation modification site on TMBIM6 mRNA. When performing qRT-PCR, the amount of each sample added was 1 μl, and MazF- was used as a control. MazF-correction formula: % MazF- - = (2 ^–CtMazF +^)/ (2 ^–CtMazF -^)100%.

### Bioinformatics analysis

The KMplot program (http://kmplot.com/analysis/) and the GEPIA (http://gepia.cancer-pku.cn/) database were used to plot the Kaplan-Meier survival curves of RBM15, TMBIM6, and IGF2BP3. The plot of the Pearson Correlation Coefficient (RBM15/TMBIM6 and TMBIM/IGF2BP3) and the differential expression boxplot in HNSC were based on the GEPIA datasets.

### Hoechst staining assay

The apoptosis assay was performed 48 h after transfection. The clean coverslip was immersed in 70% ethanol for 5 min, washed with sterile PBS three times, and then washed with a cell culture solution again. The coverslip was placed in a six-well plate; transfected cell suspension was added and incubated overnight. 0.5 ml of fixative was added and was fixed for 10 min, after which it was removed and washed twice with PBS for 3 min each time. 0.5 ml Hoechst 33258 staining solution was added and stained for 5 min, after which the staining solution was removed, washed twice with PBS, and the liquid was drained. Cell apoptosis was detected at 350 nm by a fluorescence microscope.

### RNA stability assays

To measure the stability of TMBIM6 mRNA in LSCC under the influence of knockdown or overexpression of RBM15/IGF2BP3, actinomycin D (a9415; Sigma, USA, 5 μg/mL) was applied to cells. The procedure of isolating total RNA for qPCR analysis was as described previously. Finally, the mRNA expression at the specified time was calculated and normalized using GAPDH.

### Statistical analysis

Statistical analyses were performed using SPSS version 17.0 software. The graphics were mainly plotted by GraphPad Prism 7.0. The statistically significant differences were evaluated by the two-tailed Student’s t-test. The OS analysis of LSCC patients was plotted by the Kaplan-Meier method and the log-rank test. The correlations among RBM15, TMBIM6, and IGF2BP3 expression in LSCC patients were calculated by Pearson correlation analysis. A *p*-value of < 0.05 was considered statistically significant.

## Results

### RBM15 is overexpressed in LSCC and associated with poor prognosis

**Fig. 1:**
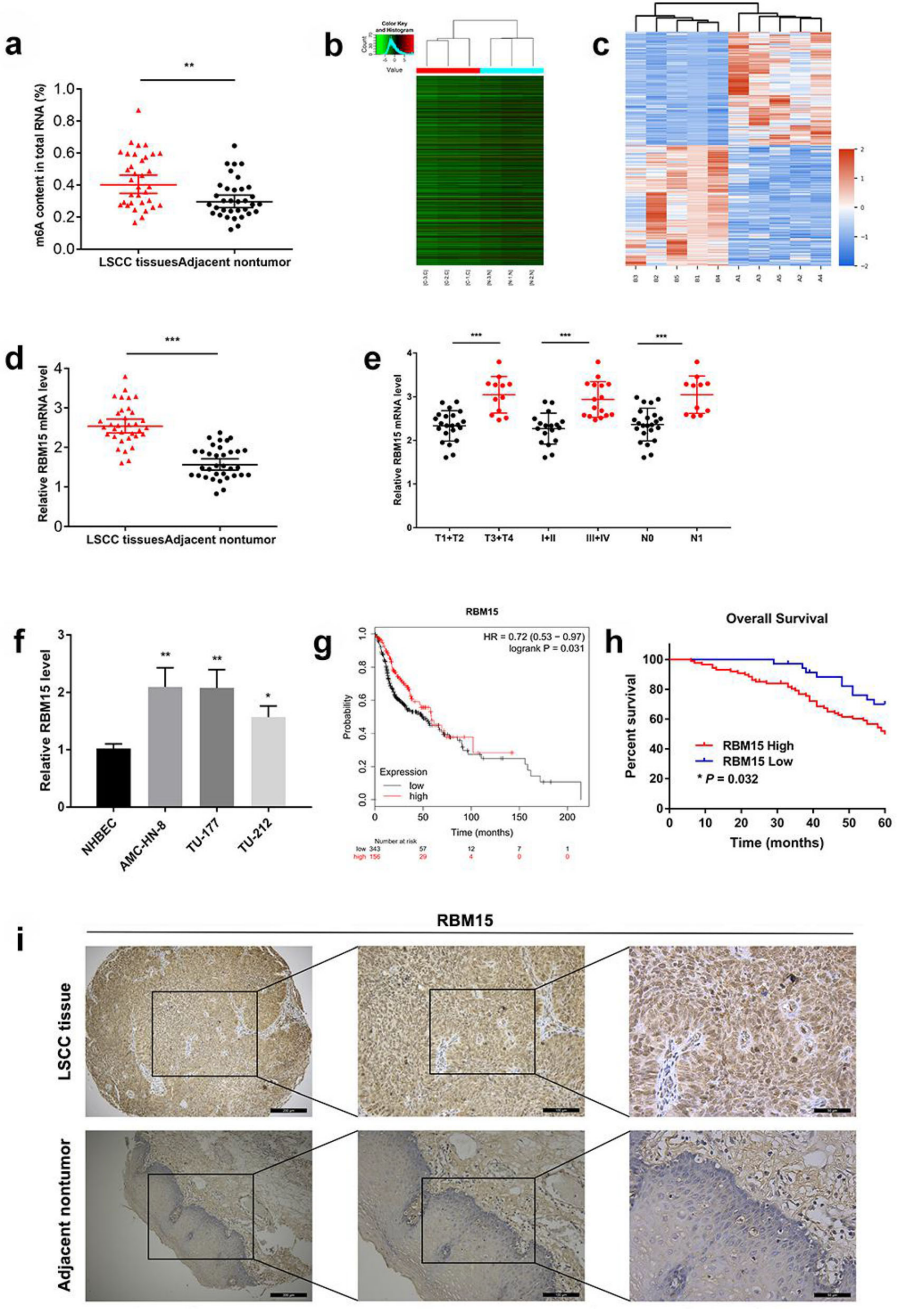
RBM15 was overexpressed in LSCC and associated with poor prognosis. **a** Global RNA m6A methylation was evaluated by the m6A RNA Methylation Quantification Kit. **b** Heatmap of differentially expressed genes in LSCC identified by mRNA-seq. **c** Protein Cluster Analysis heatmap of significantly differentially expressed proteins in LSCC tissues and adjacent nontumor tissue. **d** qRT-PCR assay verified the expression of RBM15 in 34 pairs of LSCC specimens. **e** The expression level of RBM15 was related to the clinicopathological characteristics of LSCC patients. **f** Expression levels of RBM15 in NHBEC and LSCC cell lines were examined using RT-qPCR. **g** Kaplan–Meier analysis of Overall Survival in HNSC patients. **h** Kaplan-Meier survival analysis of 122 LSCC tumour specimens showed that high RBM15 expression levels were significantly associated with poor OS. **i** Representative image of IHC staining of RBM15 in 122 LSCC tumour specimens

To investigate the roles of the m6A-associated genes in LSCC development, we first analyzed the methylation level in 34 LSCC and paired standard samples. We found that the m6A RNA modification in LSCC tissues was increased (Fig. 1a). The hierarchical clustering results showed that there were systematic differences in mRNA expression levels between the 3 pairs of laryngeal carcinoma and adjacent tissues. The screening conditions were restricted to the threshold of absolute fold change greater than 1 and *p*-value less than 0.05. The results showed that compared with adjacent non-tumor tissues, there were 841 differentially expressed mRNAs in LSCC (Fig. 1b). Protein Cluster Analysis was performed on 5 pairs of LSCC tissues and adjacent non-tumour tissues. The results showed that compared with adjacent non-tumor tissues, there were 1826 proteins differentially expressed in LSCC, of which 885 were up-regulated, and 941 were down-regulated (Additional file 2: Figure S1). Combining with the proteomics results of laryngeal cancer tissue, the RBM15 that were differentially expressed in m6A methyltransferase “writers” was screened out as the most significant (Fig. 1c). Our data revealed a significantly higher RBM15 mRNA level in the LSCC compared to the non-tumorous tissues (Fig. 1d). Moreover, the qRT-PCR results in 34 pairs of LSCC tissues suggested that the expression level of RBM15 was associated with the clinicopathological characteristics of LSCC. T3 + T4 tumours, III + IV tumours, and N1 tumours had higher expression levels of RBM15 (Fig. 1e). The expression of RBM15 was confirmed in 3 LSCC cell lines, namely TU-212, TU-177, AMC-HN-8, and normal human bronchial epithelial cells. The results suggested that in these measured laryngeal cancer cell lines, the expression level of RBM15 was more significant than in NHBEC cells (Fig. 1f). Analysis of TCGA survival data indicated that among HNSC patients, patients with low RBM15 expression had a longer median survival time (Fig. 1g). Furthermore, IHC assays were performed to determine the localization of RBM15 in LSCC tissue and its expression in 122 pairs of matched cancer tissues and adjacent non-cancerous tissues. As shown in Fig. 1i, RBM15 was mainly located in the nucleus in LSCC tissues, and the expression level in cancer tissues was significantly higher than that in adjacent non-tumor tissues. These results implied that RBM15 was overexpressed in LSCC tissues. Besides, the 60-month follow-up results showed that the overexpression of RBM15 was closely related to the poor prognosis of LSCC patients (Fig. 1h).

### RBM15 promotes LSCC cells migration and invasion in vitro and in vivo

**Fig. 2:**
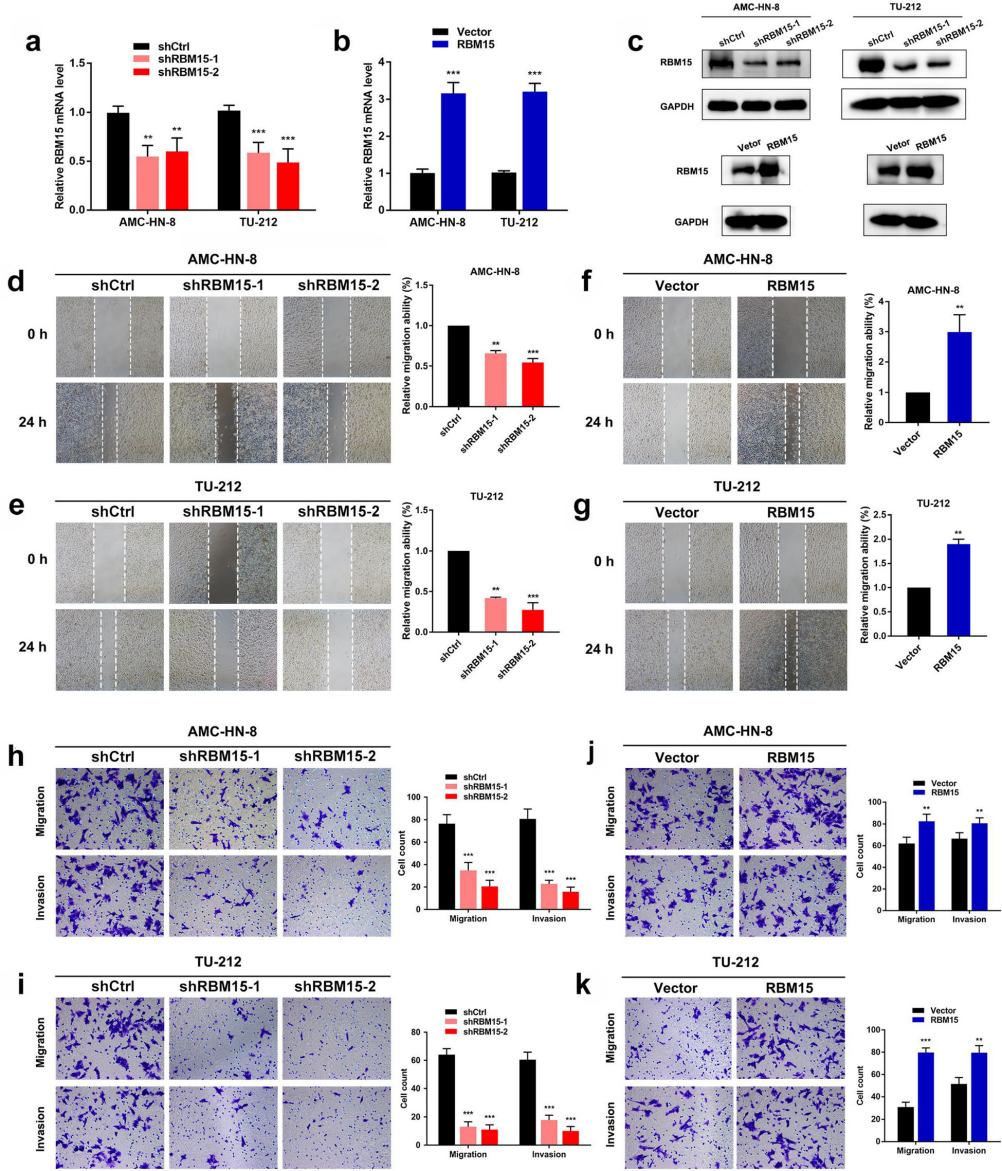
Interference of the expression of RBM15 negatively affected migration and invasion in LSCC cells. **a** The expression efficiency of RBM15 after transfection with shCtrl or shRBM15 in LSCC cells. **b** The expression efficiency of RBM15 after transfection with Vector or RBM15 in LSCC cells. **c** Western blot analysis to measure RBM15 protein levels in LSCC cells transfected with shCtrl and/or shRBM15 and/or RBM15 vectors. **d**-**g** Wound healing assays were performed to measure the cell migration ability in LSCC cells after transfection with shCtrl and/or shRBM15 and/or RBM15 vectors. **h**-**k** Transwell assays were conducted to examine the cell migration and invasion ability in LSCC cells after transfection with shCtrl and/or shRBM15 and/or RBM15 vectors

In order to explore the potential function of RBM15 in LSCC, we synthesized RBM15 specific shRNA to obstruct the expression of endogenous RBM15 in TU-212 and AMC-HN-8 cell lines. RBM15 expression was reduced after shRBM15 transfection. After transfection with overexpressing RBM15 lentivirus, the expression of RBM15 increased (Fig. 2a, b). Similarly, qRT-PCR results were confirmed at the protein level (Fig. 2c). CCK-8 assays showed that RBM15 knockdown significantly repressed cell proliferation of LSCC cells, while RBM15 overexpression markedly promotes cell proliferation (Additional file 9: Figure S7). After knocking down RBM15, the migration ability of TU-212 and AMC-HN-8 cells obviously decreased (Fig. 2d, e). RBM15 overexpression apparently increased the migration speed of TU-212 and AMC-HN-8 cells (Fig. 2f, g). The transwell assays demonstrated that the migration and invasion ability of LSCC cells decreased when transfected with shRNA against RBM15 compared with the control group (Fig. 2h, i). On the contrary, when RBM15 was overexpressed, the migration and invasion ability of LSCC cells significantly increased (Fig. 2j, k).

**Fig. 3:**
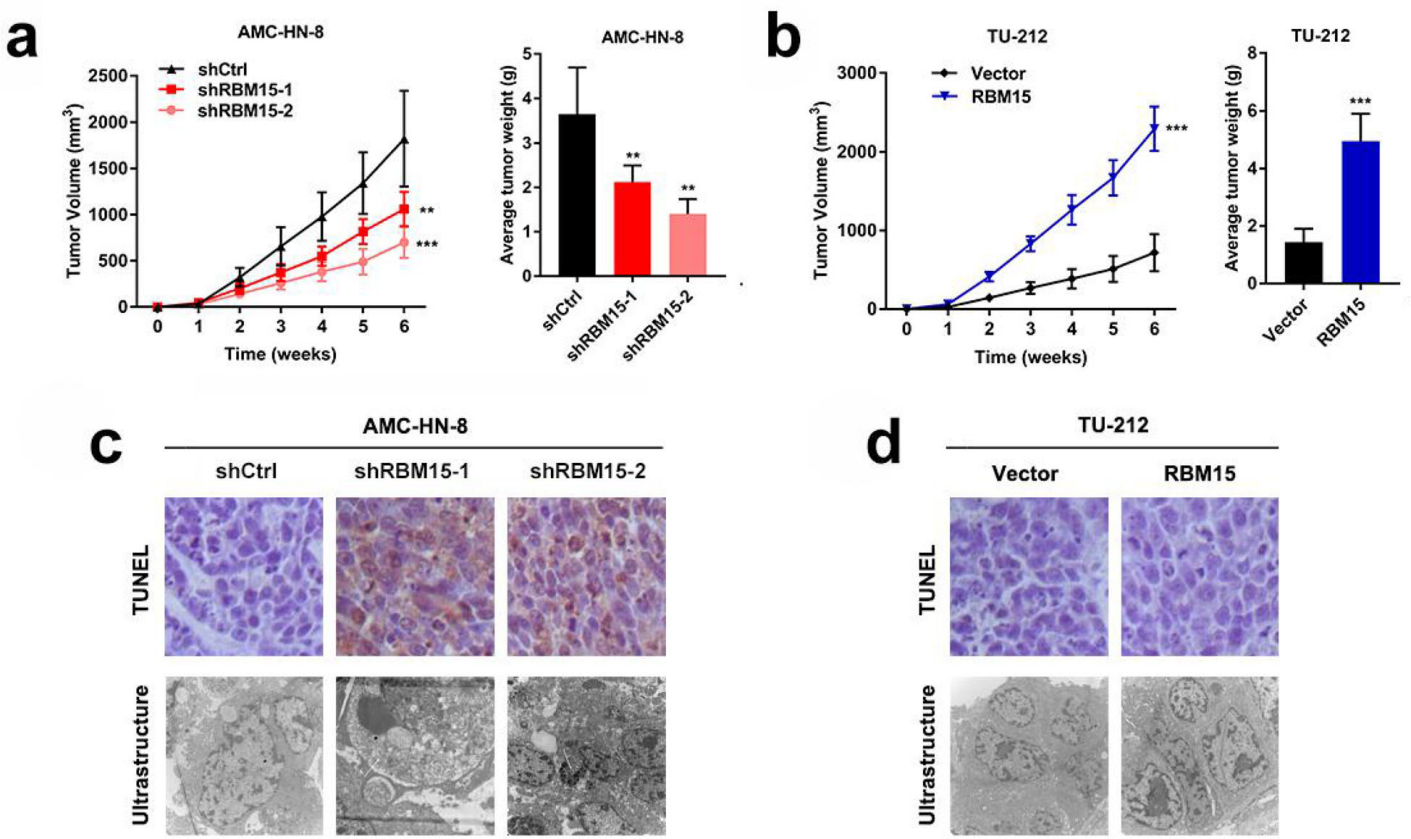
RBM15 regulates LSCC growth and apoptosis in mice. **a** Inhibition of RBM15 impaired the growth of xenografted tumors. **b** Overexpression of RBM15 stimulated the growth of xenografted tumors. **c** and **d** Representative images of TUNEL staining quantification of RBM15 positive staining in xenografted tumours after transfection with shCtrl and/or shRBM15 and/or RBM15 vectors

To further evaluate the effects of RBM15 on the regulation of LSCC cells progression in vivo, LSCC cells were subcutaneously injected into 30 mice. However, as expected, when AMC-HN-8 cells with down-regulated RBM15 expression were injected, the xenograft tumour volume was significantly reduced compared to the control group (Fig. 3a). The tumor volumes in the RBM15 overexpression group were increased compared to those in the vector group (Fig. 3b). Additionally, we performed TUNEL staining and transmission electron microscopy to evaluate cell apoptosis. The typical apoptotic morphology was observed in the RBM15 gene knockout group, while the control group, vector, and RBM15 overexpression group showed a complete cell membrane and complete organelle morphology (Fig. 3c, d). In summary, the above results emphasized the crucial role of RBM15 in the progression of LSCC in vitro and in vivo.

### RBM15 participates in the methylation of TMBIM6 in the form of m6A modification

**Fig. 4:**
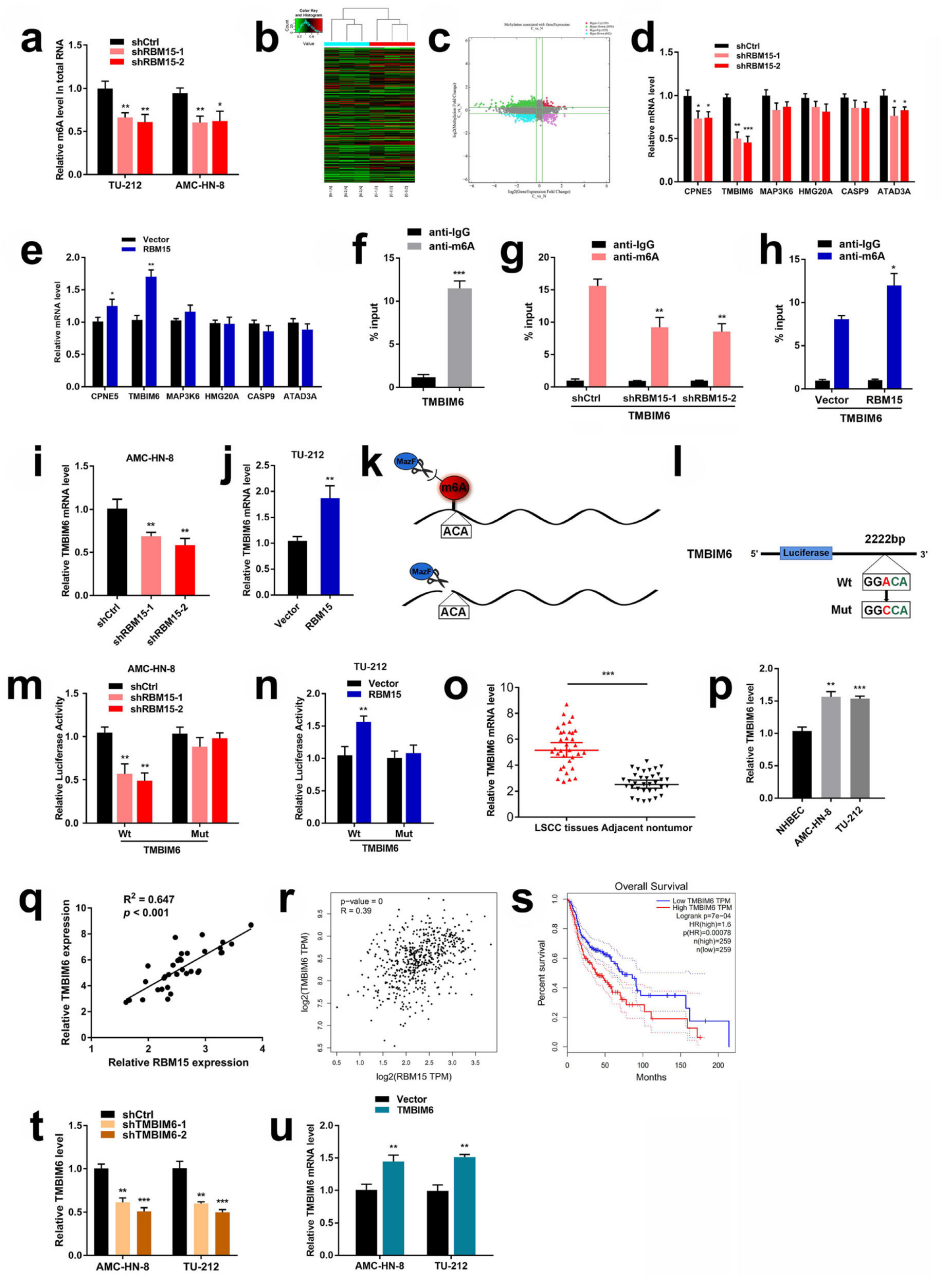
RBM15 participated in the methylation of TMBIM6 in the form of m6A modification. **a** The methylated RNA (m6A) level was determined after RBM15 knockdown in the LSCC cell lines. **b** Hierarchical clustering for mRNAs with differential “m6A quantity”. **c** Coalition analysis of significant changes in RNA expression level and m6A level in pairs of LSCC specimens. **d** The expression of selected mRNAs was investigated by qRT-PCR in AMC-HN-8 cells transfected with shCtrl or shRBM15. **e** The expression of selected mRNAs was investigated by qRT-PCR in TU-212 cells transfected with vector or RBM15-expressing plasmid. **f** MeRIP assay was performed to identify whether m6A modification was enriched in the TMBIM6 sequence. **g** and **h** MeRIP assays were performed to identify variation in m6A modification enrichment in TMBIM6 after silencing or overexpressing RBM15 in LSCC cells. **i** and **j** qRT-PCR analysis of TMBIM6 mRNA after RBM15 inhibition or overexpression. **k** Diagram summarizing the mechanism of MazF RNA endonuclease. MazF cleaves only nonmethylated RNA, sites with m6A methylation remained uncleaved. **l** A schematic diagram showing the methylation site on TMBIM6 and mutates this site. **m** The luciferase activities in AMC-HN-8 cells co-transfected with TMBIM6-WT or TMBIM6-Mut together with shCtrl or shRBM15. **n** The luciferase activities in TU-212 cells co-transfected with TMBIM6-WT or TMBIM6-Mut together with Vector or RBM15. **o** and **p** qRT-PCR verified the expression of TMBIM6 in 34 pairs of LSCC tissues and cell lines. **q** Correlation analysis was conducted on the expression levels of RBM15 and TMBIM6 in 34 cases of LSCC tissues. **r** Correlation analysis was performed on the expression levels of RBM15 and TMBIM6 in the TCGA database. **s** The analysis results of the TCGA database showed the relationship between the expression level of TMBIM6 in HNSC patients and the overall survival rate. **t** The expression efficiency of TMBIM6 after transfection with shCtrl or shTMBIM6 in LSCC cells. **u** The expression efficiency of TMBIM6 after transfection with Vector or TMBIM6 in LSCC cells

In order to investigate the molecular mechanism of RBM15 regulating LSCC m6A modification, LSCC cells were transfected with shRBM15. As shown in the results of the m6A RNA Methylation Quantification Kit (ab185912, Abcam, UK), the m6A methylation level in LSCC cells significantly decreased after inhibiting RBM15 (Fig. 4a). First, m6A RNA immunoprecipitation (RIP) microarray was performed to identify candidate genes with high m6A methylation modification and increased mRNA expression (Fig. 4b, c). The top 6 mRNAs were screened based on fold change and *p*-value (Additional file 3: Table S2). Moreover, LSCC cells were transfected with shRBM15 lentivirus for 48 h, and the qRT-PCR data indicated that the mRNA levels of CPNE5, TMBIM6, and ATAD3A decreased after RBM15 knockdown. In contrast, the mRNA levels of CPNE5 and TMBIM6 in LSCC cells in the RBM15 overexpression group showed an increasing trend (Fig. 4d, e).

Following the analysis of the above results, TMBIM6, which had the most remarkable effect of methylation mediated by RBM15, was selected for further research. MeRIP assay was performed to investigate the m6A modification status of TMBIM6 further. Our results suggest that the TMBIM6 sequence is enriched in m6A modifications (Fig. 4f). Next, we performed anti-m6A immunoprecipitation followed by qRT-PCR analysis of the m6A immunoprecipitated RNAs. The results showed that the down-regulation of RBM15 caused a decrease in the m6A modification of the TMBIM6 mRNA. By contrast, RBM15 overexpression increased m6A modification of the TMBIM6 mRNA (Fig. 4g, h). From the perspective of TMBIM6 mRNA, when RBM15 was knocked down or overexpressed, the mRNA level of TMBIM6 was down-regulated or up-regulated, respectively (Fig. 4i, j). These results fully illustrate the regulation of TMBIM6 expression by RBM15.

Next, the bacterial single-stranded RNase MazF assay was used to identify the sites of m6A methylation. This RNase MazF could digest ACA sites of cellular RNAs without m6A methylation, while sites with m6A methylation remained uncleaved (Fig. 4k). A potential m6A motif was identified as the m6A deposition site of TMBIM6 downstream, located at base 2222 of the 3’UTR, which was consistent with the prediction of the SRAMP website. We mutated ACA in the base sequence to CCA and then performed a luciferase assay (Fig. 4l). Dual-luciferase reporter assay was further used to determine the combinations of RBM15 and TMBIM6. The results indicated that the luciferase activity in the shRBM15 group was significantly lower compared to shCtrl group, while the activity of the TMBIM6-Mut group did not significantly change (Fig. 4m). Similarly, in TU-212 cells, the luciferase activity of the RBM15 overexpression group was significantly increased compared to the vector group, while the activity of the TMBIM6-Mut group did not significantly change (Fig. 4n). These results suggested a direct interaction between TMBIM6 and RBM15.

TMBIM6 was determined as a direct target of RBM15, and qRT-PCR was performed in the 34 pairs of LSCC tissues. TMBIM6 was remarkably upregulated in LSCC tissues compared to adjacent nontumor tissues. Compared with the NHBEC cell line, the TMBIM6 mRNA level in the LSCC cell line also increased (Fig. 4o, p). IHC was performed to detect the expression of TMBIM6 in 122 pairs of LSCC samples. As shown in Additional file 4: Figure S2, high expression of TMBIM6 was detected in cancerous tissues. Kaplan-Meier survival curves indicated that patients with high TMBIM6 expression had poorer overall survival than those with low TMBIM6 expression (Additional file 5: Figure S3). By analyzing the information of 34 specimens, it was revealed that RBM15 mRNA expression in LSCC tissues was positively associated with TMBIM6 levels (Fig. 4q), which was consistent with the TCGA database (Fig. 4r). By analyzing the TCGA database, the Kaplan–Meier survival curve showed that the overall survival rate of HNSC patients with high TMBIM6 expression was worse than the prognosis of low expression (Fig. 4s). To further facilitate research, we constructed a lentivirus that knockdown and overexpressed TMBIM6 (Fig. 4t, u).

### RBM15 accelerates LSCC malignant progression by upregulating TMBIM6

**Fig. 5:**
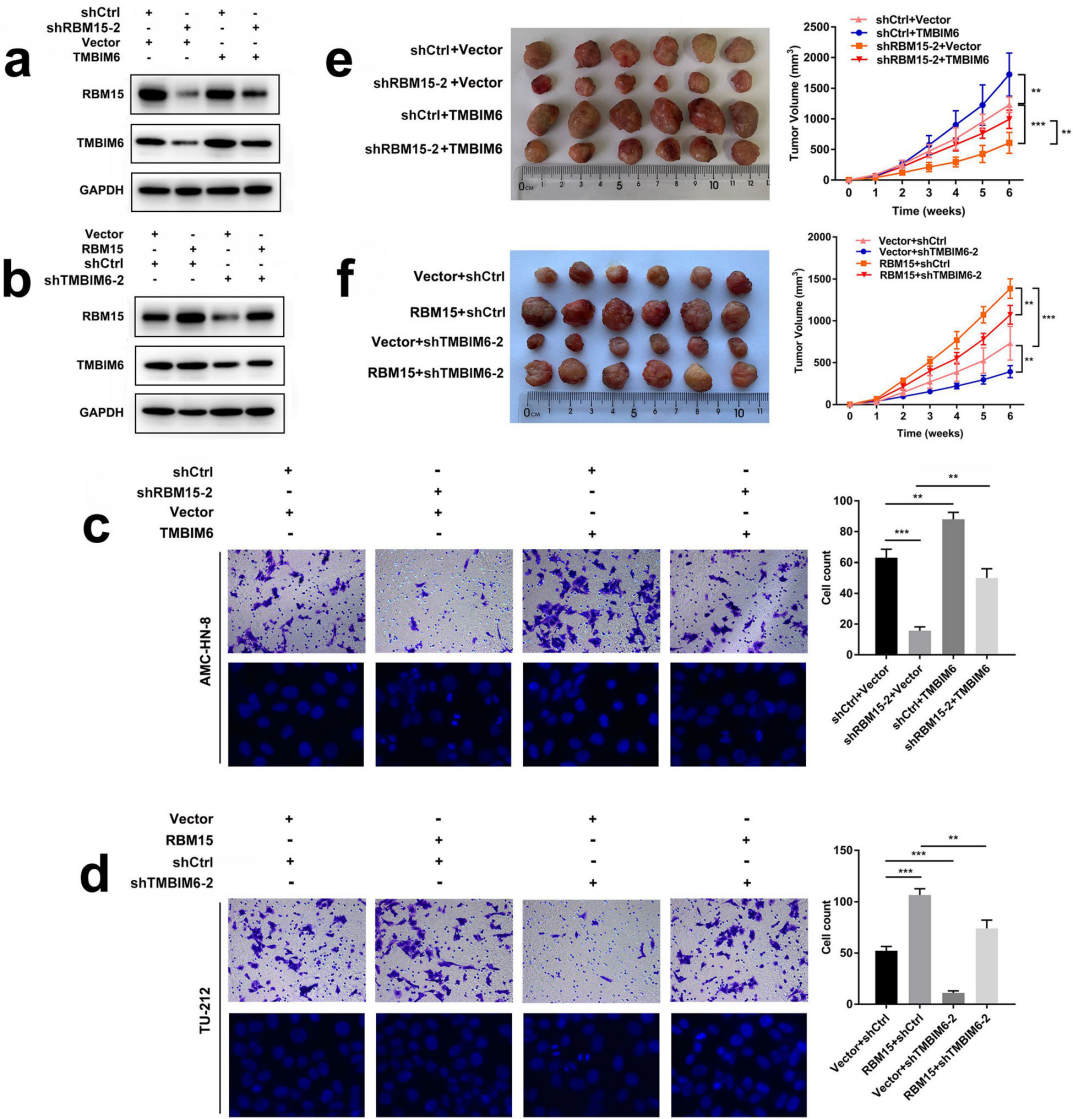
RBM15 accelerates LSCC malignant progression by upregulating TMBIM6. **a** Western blot was performed to investigate the expression of RBM15 and TMBIM6 in AMC-HN-8 cells after transfection with shRBM15–2 and/or TMBIM6. **b** Western blot was conducted to examine the expression of RBM15 and TMBIM6 proteins in TU-212 cells after transfection with shTMBIM6–2 and/or RBM15. **c** Transwell assay was performed to measure the cell invasion ability of AMC-HN-8 cells after transfection with shRBM15–2 and/or TMBIM6. Hoechst staining assay was performed to measure cell apoptosis of AMC-HN-8 cells after transfection with shRBM15–2 and/or TMBIM6. **d** Transwell assay was performed to measure the cell invasion ability of TU-212 cells after transfection with shTMBIM6–2 and/or RBM15. Hoechst staining assay was performed to measure the cell apoptosis of TU-212 cells after transfection with shTMBIM6–2 and/or RBM15. **e** Knockdown of RBM15 inhibited TMBIM6-induced AMC-HN-8 cells subcutaneously tumor growth in nude mice. **f** Knockdown of TMBIM6 inhibited RBM15-induced TU-212 cells subcutaneously tumor growth in nude mice

The ablation of RBM15 could partially neutralize the effects of increased TMBIM6 on the expression of TMBIM6 in AMC-HN-8 cells (Fig. 5a). In TU-212 cells, when RBM15 was overexpressed, the protein expression of RBM15 and TMBIM6 increased. The overexpression of RBM15 could partially neutralize the effect of inhibiting TMBIM6 on TMBIM6 protein expression (Fig. 5b). Moreover, transwell assays confirmed that the invasion ability of AMC-HN-8 cells was significantly reduced after inhibiting the expression of RBM15. The reduction of invasive ability of LSCC cells exerted by shRBM15–2 was reversed by co-transfection with overexpression TMBIM6. The inhibition of RBM15 resulted in obviously increased cell apoptosis compared with those in the control group, characterized by brightly stained nuclei, nuclear condensation, and fragmentation. Yet, the overexpression of TMBIM6 partially neutralized the ablation effect of RBM15 (Fig. 5c). Transwell assays confirmed that the invasive ability of TU-212 cells was significantly reduced after inhibiting the expression of TMBIM6. The reduction of the invasion ability of LSCC cells exerted by shTMBIM6–2 was reversed by co-transfection with overexpression of RBM15. Besides, the inhibition of TMBIM6 resulted in obviously increased cell apoptosis compared with the control group; however, the overexpression of RBM15 partially neutralized the ablation effect of TMBIM6 (Fig. 5d). Consistently, nude mice xenografts injected with the AMC-HN-8 cells revealed that knockdown of RBM15 counteracted the effects of increased TMBIM6 on tumor volumes (Fig. 5e). Nude mice xenografts injected with the TU-212 cells revealed that knockdown of TMBIM6 counteracted the effects of increased RBM15 on tumor volumes (Fig. 5f).

In brief, the m6A modification on TMBIM6 that includes RBM15 is essential for the up-regulation of TMBIM6 to promote the progression and invasion of LSCC.

### RBM15-mediated m6A modification of TMBIM6 mRNA enhances TMBIM6 stability through IGF2BP3-dependent

**Fig. 6:**
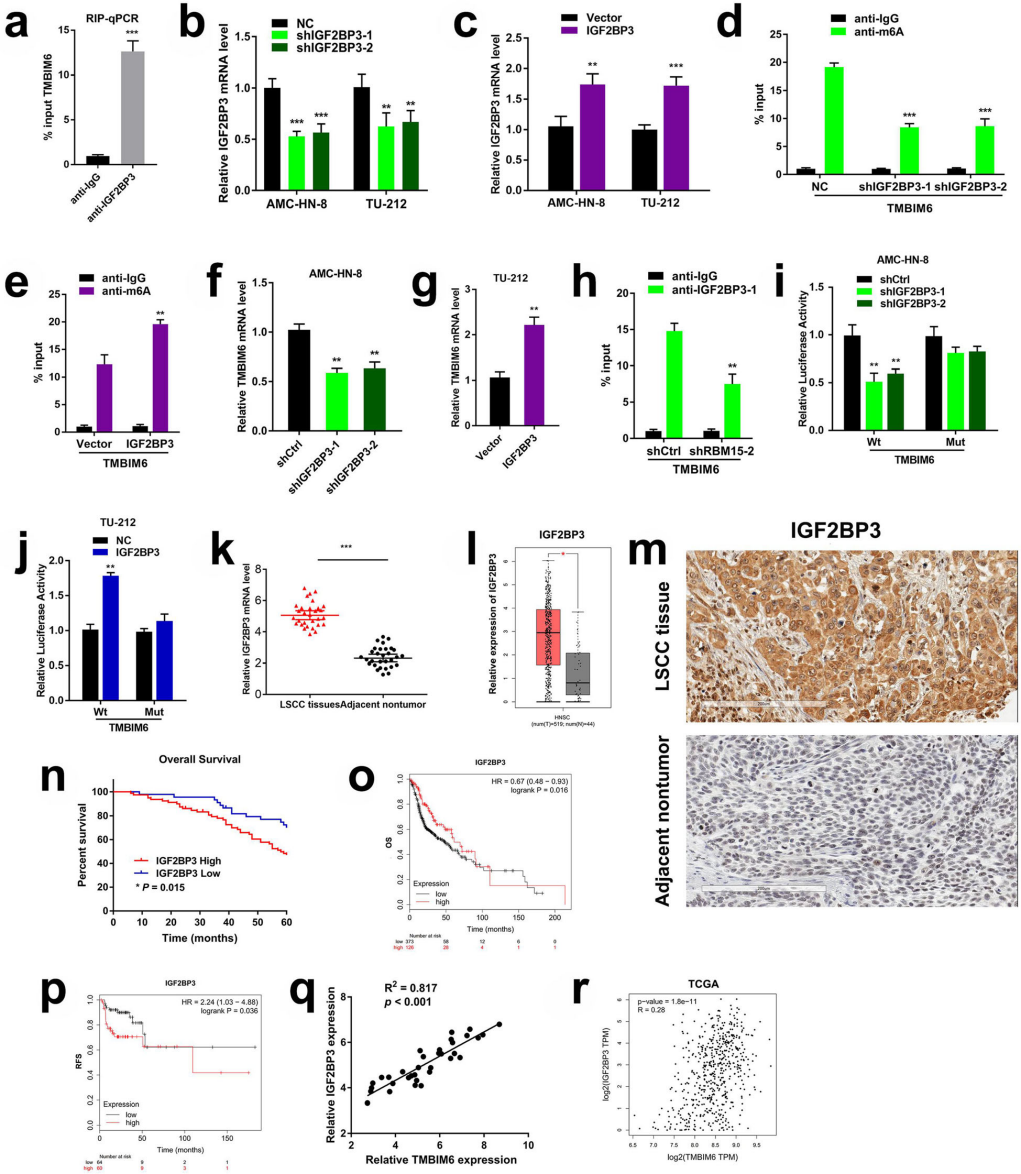
IGF2BP3 was involved in m6A methylation modification in LSCC. **a** RIP-qPCR was showing the enrichment of IGF2BP3 binding to TMBIM6 m6A modification sites. **b** and **c** After knocked down or overexpressed IGF2BP3, qRT-PCR evaluated the expression of IGF2BP3 in LSCC cells. **d** and **e** MeRIP assays were performed to identify variation in m6A modification enrichment in TMBIM6 after silencing or overexpressing IGF2BP3 in LSCC cells. **f** and **g** qRT-PCR of TMBIM6 mRNA after IGF2BP3 inhibition or overexpression in LSCC cells. **h** RIP-qPCR unveiled the interaction within IGF2BP3 and TMBIM6 mRNA after RBM15 inhibition. **i** The luciferase activities in AMC-HN-8 cells co-transfected with TMBIM6-WT or TMBIM6-Mut together with shCtrl or shIGF2BP3. **j** The luciferase activities in TU-212 cells co-transfected with TMBIM6-WT or TMBIM6-Mut together with Vector or IGF2BP3. **k** qRT-PCR was used to detect IGF2BP3 expression in 34 pairs of LSCC tissues. **l** Results based on the TCGA and GEPIA databases showed the expression level of IGF2BP3 in HNSC. **m** IHC staining of IGF2BP3 in LSCC tumour specimens. **n** Kaplan-Meier survival analysis showed that the expression level of IGF2BP3 in LSCC patients was significantly related to the OS. **o** and **p** Data from the TCGA database showed that the expression level of IGF2BP3 in HNSC patients was significantly related to OS and RFS. **q** Correlation analysis was conducted on the expression levels of TMBIM6 and IGF2BP3 in 34 cases of LSCC tissues. **r** Correlation analysis was performed on the expression levels of TMBIM6 and IGF2BP3 in the TCGA database

Given that the m6A methylation modification level of TMBIM6 and its mRNA expression level are simultaneously increased, we speculate that m6A modification enhances the stability of TMBIM6 through the IGF2BP family dependence. Combined with our data, the mRNA-seq results showed prominent differential expression of IGF2BP3, and the proteomics data revealed that the expression of IGF2BP2 and IGF2BP3 was increased, and the differential expression of IGF2BP3 was particularly significant. Therefore, we verified whether IGF2BP2 or IGF2BP3 were m6A-dependent in a manner that enhances the stability of TMBIM6. Firstly, we detected the enrichment of IGF2BP3 binding to TMBIM6 m6A modification sites by RNA RIP-qPCR assay (Fig. 6a), after which designed and synthesized knockdown and overexpression lentiviral vectors for IGF2BP2 and IGF2BP3, respectively (Fig. 6b, c and Additional file 6: Figure S4). Interestingly, the anti-m6A immunoprecipitation followed by qRT-PCR analysis results showed that the inhibition of IGF2BP3 caused a decrease in the m6A modification of TMBIM6 mRNA. By contrast, IGF2BP3 overexpression increased m6A modification of the TMBIM6 mRNA (Fig. 6d, e).

From the perspective of TMBIM6 mRNA, when IGF2BP3 was knocked down or overexpressed, the mRNA level of TMBIM6 was down-regulated or up-regulated, respectively (Fig. 6f, g). Nevertheless, when IGF2BP2 was knocked-down or overexpressed, it had a trifling impact on TMBIM6 expression (Additional file 7: Figure S5). Furthermore, RIP-qPCR revealed that the ablation of RBM15 could weaken the direct interaction within IGF2BP3 and TMBIM6 mRNA in LSCC cells. This result indicated that RBM15, IGF2BP3, and TMBIM6 mRNA were strongly interrelated (Fig. 6h).

A luciferase reporter assay was performed to further reveal weather TMBIM6 levels were regulated by IGF2BP3 involved in m6A modification. The results indicated that the luciferase activity of the shIGF2BP3 group was significantly lower than that of the shCtrl group, while the activity of the TMBIM6-Mut group did not significantly change. Mutually, the luciferase activity of the IGF2BP3 overexpression group was significantly increased compared to the vector group, while the activity of the TMBIM6-Mut group did not significantly change (Fig. 6i, j). In summary, these data revealed that IGF2BP3 affected the stability of TMBIM6 by participating in the TMBIM6 m6A modification.

Subsequently, qRT-PCR was applied to investigate the IGF2BP3 expression in LSCC, and the detection results were generally consistent with the TCGA database, i.e., IGF2BP3 in LSCC was significantly upregulated compared to healthy tissues adjacent to cancer (Fig. 6k, l). Moreover, immunohistochemical analysis was carried out to detect the expression of IGF2BP3 in LSCC. As shown in Fig. 6m, stronger IGF2BP3 staining was detected in 122 LSCC compared with adjacent non-tumor tissues. The 60-month follow-up results showed that the overexpression of IGF2BP3 was closely related to the poor prognosis of LSCC patients (Fig. 6n). Furthermore, TCGA data indicated that HNSC patients with high expression of IGF2BP3 had a poor prognosis (*p* < 0.05; Fig. 6o, p). Besides, a prominent positive correlation between TMBIM6 and IGF2BP3 was calculated using RT-PCR quantitation. Additional data obtained from the TCGA database were shown to be consistent with our qRT-PCR results (Fig. 6q, r). Also, the qRT-PCR results in 34 pairs of LSCC tissues suggested that the IGF2BP3 expression was associated with the clinicopathological characteristics of LSCC. T3 + T4 tumours, III + IV tumours, and N1 tumours had higher expression levels of IGF2BP3 (Additional file 8: Figure S6).

**Fig. 7:**
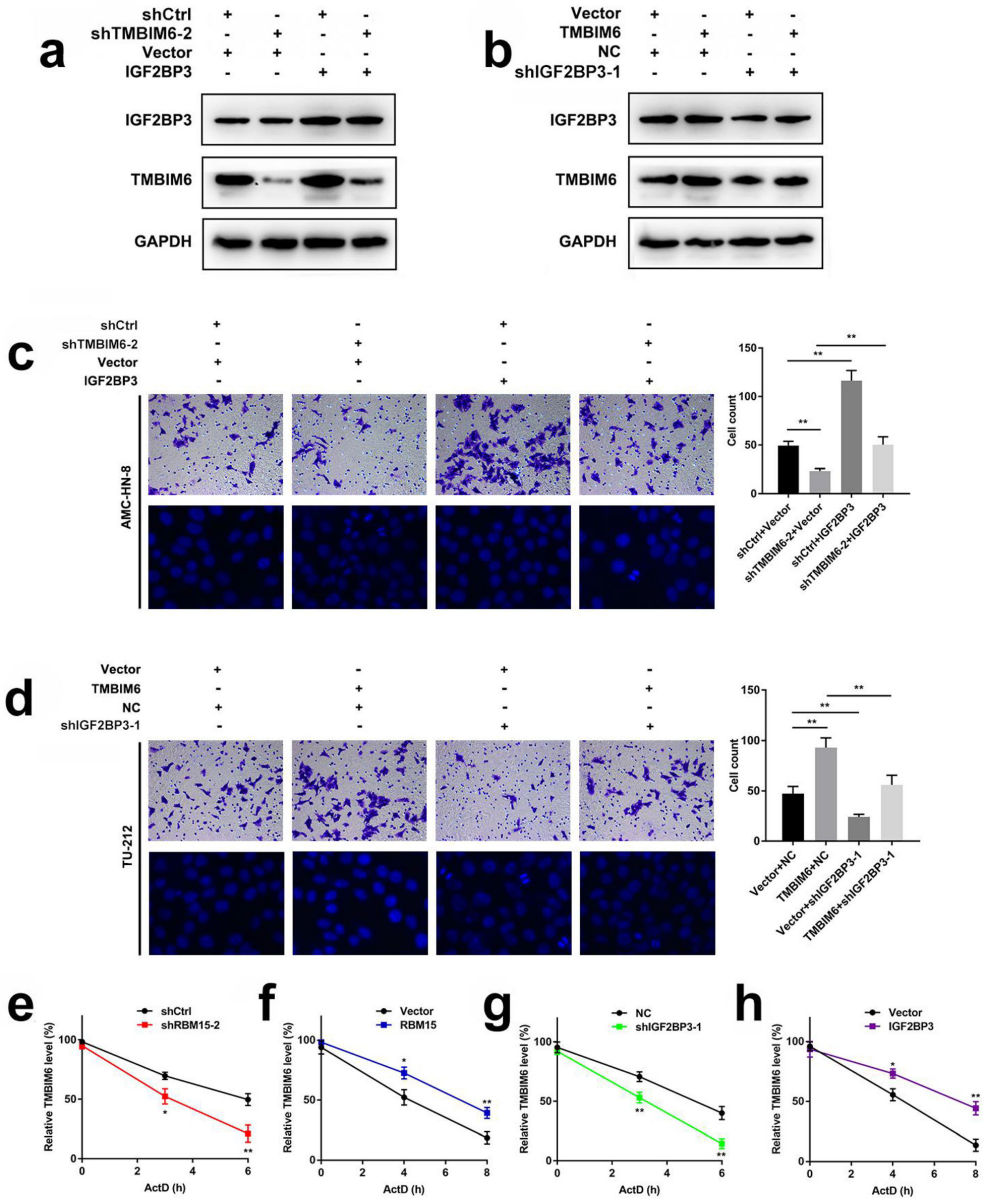
m6A modification of TMBIM6 mRNA enhances TMBIM6 stability through IGF2BP3-dependent. **a** Western blot was performed to investigate the expression of TMBIM6 and IGF2BP3 in AMC-HN-8 cells after transfection with shTMBIM6–2 and/or IGF2BP3. **b** Western blot was conducted to examine the expression of TMBIM6 and IGF2BP3 in TU-212 cells after transfection with shIGF2BP3–1 and/or TMBIM6. **c** Transwell assay was performed to measure the cell invasion ability of AMC-HN-8 cells after transfection with shTMBIM6–2 and/or IGF2BP3. Hoechst staining assay was performed to measure cell apoptosis of AMC-HN-8 cells after transfection with shTMBIM6–2 and/or IGF2BP3. **d** Transwell assay was performed to measure the cell invasion ability of TU-212 cells after transfection with shIGF2BP3–1 and/or TMBIM6. Hoechst staining assay was performed to measure cell apoptosis of TU-212 cells after transfection with shIGF2BP3–1 and/or TMBIM6. **e**-**h** RNA stability assay showed that different transfection groups have a different effect on the half-life of TMBIM6 mRNA

To determine whether IGF2BP3 is an important influence factor of TMBIM6 in LSCC tumorigenesis, a rescue experiment was performed. As shown in Fig. 7a, the ablation of TMBIM6 could partially neutralize the effects of increased IGF2BP3 on the expression of TMBIM6 in AMC-HN-8 cells. In TU-212 cells, overexpression of TMBIM6 had a trifling effect on the expression level of IGF2BP3 protein, and the expression of TMBIM6 protein also decreased after IGF2BP3 was knocked down. Nevertheless, the overexpression of TMBIM6 could partially neutralize the effect of inhibiting IGF2BP3 on TMBIM6 protein expression (Fig. 7b). Moreover, transwell assays confirmed that the invasion ability of AMC-HN-8 cells was significantly reduced after inhibiting the expression of TMBIM6. Reduced invasive ability of LSCC cells exerted by shTMBIM6–2 was reversed by co-transfection with overexpression of IGF2BP3. The ablation of TMBIM6 resulted in obviously increased cell apoptosis compared with the control group and was characterized by brightly stained nuclei, nuclear condensation, and fragmentation. However, the overexpression of IGF2BP3 partially neutralized the ablation effect of TMBIM6 (Fig. 7c). Transwell assays confirmed that the invasion ability of TU-212 cells was significantly reduced after inhibiting the expression of IGF2BP3. Reduced invasion ability of LSCC cells exerted by shIGF2BP3–1 was reversed by co-transfection with overexpression of TMBIM6. In addition, the inhibition of IGF2BP3 resulted in obviously increased cell apoptosis compared with the control group; however, the overexpression of TMBIM6 partially neutralized the ablation effect of IGF2BP3 (Fig. 7d). RNA stability assays suggested that the ablation of RBM15 shortened the half-life of TMBIM6 mRNA, while overexpression of RBM15 prolonged the half-life of TMBIM6 mRNA (Fig. 7e, f).

**Fig. 8:**
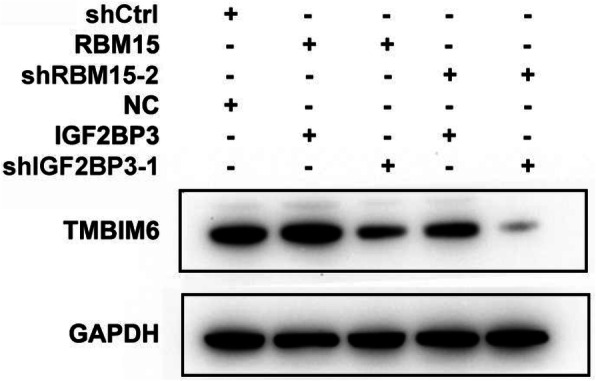
Western blot results suggested that after both RBM15 and IGF2BP3 were knocked down in AMC-HN-8 cells, the TMBIM6 protein level was significantly reduced, and the TMBIM6 protein expression increased after both RBM15 and IGF2BP3 were overexpressed, but was restored after the knockdown of RBM15 or IGF2BP3

Furthermore, the ablation of IGF2BP3 shortened the half-life of TMBIM6 mRNA. In contrast, overexpression of IGF2BP3 prolonged the half-life of TMBIM6 mRNA (Fig. 7g, h). To further confirm whether RBM15 and IGF2BP3 were involved in regulating the expression of TMBIM6, another rescue experiment was conducted. In AMC-HN-8 cells with simultaneous inhibition of RBM15 and IGF2BP3, the expression of TMBIM6 was significantly reduced (Compared lane 1 to lane 5, Fig. 8). In contrast, while the simultaneous overexpression of RBM15 and IGF2BP3 increased TMBIM6 expression, downregulation of RBM15 or IGF2BP3 could impact TMBIM6 overexpression (compared lane 2, 3 to lane 4, Fig. 8).

Taken together, the above results reconfirmed that RBM15 and IGF2BP3 had an essential role in promoting the stability of TMBIM6.

## Discussion

There are many types of chemical modifications in RNA. Among them, m6A is the most abundant and reversible in mammalian mRNA and non-coding RNA [20, 21]. The m6A modification has an essential role in various biological processes such as modulation of mRNA stability, pre-mRNA splicing, translation, and DNA damage repair [22, 23]. A series of researches have shown that m6A modification is related to a variety of human cancers, including hepatocellular carcinoma, gastric cancer [24, 25], glioblastoma [26], non-small cell lung cancer [27], and so on. Still, no study explored the function of m6A RNA methylation in LSCC. In the present study, our data showed that m6A modification was increased in LSCC compared with non-neoplastic tissues, which suggested that m6A may participate in the tumorigenesis.

We first found the differential m6A enrichment in RNAs of LSCC and adjacent non-neoplastic tissues by microarray. Combining proteomics results, we screened RBM15 as the most differentially expressed m6A methyltransferase in “writers”. RBM15 is a member of the SPEN (Split-end) family of proteins, which may bind to RNA by interacting with spliceosome components [28]. It is primarily localized in the nucleus. A previous study has shown that RBM15 and its paralogue RBM15B bind m6A-methylation complex to regulate XIST lncRNA m6A formation in human cells [29]. Another recent study showed that RBM15 is involved in the mechanism of mRNA methylation in the developing cortex [30].

Interestingly, RBM15 may have different roles in different types of diseases. In this study, we further revealed that the expression of RBM15 was increased compared with the non-neoplastic tissues at both mRNA and protein levels by microarray, qRT-PCR, and immunohistochemistry in LSCC. Moreover, our results indicated that the expression level of RBM15 was related to the clinicopathological characteristics and prognosis of LSCC patients. Among patients with HNSC, those with high RBM15 expression had a poor prognosis. Our data showed that inhibition of RBM15 decreased LSCC cell proliferation, migration, invasion, and apoptosis. Overexpression of RBM15 had the opposite effect, which indicates that RBM15 has a non-negligible role in LSCC and a potential value as a biomarker in the future.

We further performed m6A RNA immunoprecipitation (RIP) microarrays to identify candidate genes with high m6A methylation modification and increased mRNA expression levels. Six candidate genes were screened and verified in LSCC cell lines by using a lentiviral vector. Results showed that 6 mRNAs, including TMBIM6, were significantly hypermethylated in LSCC. By using the qRT-PCR, RIP, MazF, luciferase reporter assay, and rescue experiment, we identified TMBIM6 as the target of RBM15 in LSCC. The human TMBIM6 gene, known as Bax Inhibitor-1, is located on 12q13.12. TMBIM6 is overexpressed and has oncogene roles in multiple cancers such as squamous cervical cancer, non-small cell lung, breast, and nasopharyngeal cancers [31–34]. Yet, the effect of TMBIM6 in LSCC remains unclear. The present analysis revealed that TMBIM6, which was upregulated, exerted a cancerogenic role in LSCC. TMBIM6 downregulation markedly inhibited the migration and invasion of LSCC cells.

Moreover, the ablation of TMBIM6 increased cell apoptosis characterized by brightly stained nuclei, nuclear condensation, and fragmentation. More importantly, the overexpression of RBM15 or IGF2BP3 partially neutralized the ablation effect of TMBIM6. Furthermore, MeRIP assay identified that m6A modification was enriched in the TMBIM6 sequence. Spontaneously, RBM15 downregulation could reduce TMBIM6 expression, which suggested TMBIM6 m6A modification was an essential mechanism in the regulation of cell migration, invasion and apoptosis of LSCC.

m6A “readers” are considered as a regulator of mRNA metabolism, while mainly IGF2BPs family is associated with methylated mRNA stability [35, 36]. Among RBPs, IGF2BP3 is particularly important in tumorigenesis and tumour progression. Moreover, previous studies have addressed the molecular mechanism of IGF2BP3 carcinogenesis [37, 38]. Recent research showed that IGF2BP3 exerts a vital part in melanoma invasion and metastasis [39]. Another study suggested that upregulated IGF2BP2 may cause poor prognosis in pancreatic ductal adenocarcinoma [40]. Our data identified that the expression of IGF2BP3 was positively related to RBM15 and TMBIM6 in LSCC. We thus speculated that IGF2BP3 was involved in the process of the m6A modification of TMBIM6 by RBM15. By using MeRIP-qPCR, MazF, luciferase report assay, and rescue experiment, we revealed that IGF2BP3 regulates the m6A level of TMBIM6 mediated by RBM15. Furthermore, the downregulation of IGF2BP3 or RBM15 resulted in a significant decrease in TMBIM6 level and stability. IGF2BP3 bound to the m6A site in the 3’UTR region of TMBIM6. Moreover, IGF2BP3 controlled the stability of TMBIM6 mRNA. The Pearson Correlation Coefficient obtained from the samples data corroborated the above results, and the information obtained from the TCGA data also corroborated our results. In addition, our research also identified the expression of IGF2BP3 in laryngeal cancer. The results of the collected specimens were consistent with the results in the TCGA database. The expression of IGF2BP3 in laryngeal cancer tissues was significantly higher than that of noncancerous tissues adjacent to cancer and was associated with a poor prognosis.

## Conclusion

Overall, we proposed the vital role of RBM15-mediated m6A modification in LSCC progression. Mechanistically, as an oncogene in LSCC, RBM15 and IGF2BP3 participate in the m6A methylation modification of TMBIM6, thus regulating the expression of TMBIM6 in LSCC. We assume that RBM15 may exert an essential value in the diagnosis and treatment of LSCC in the future.

## Supplementary Information

**Additional file 1: Table S1.** The primers used in this study.

**Additional file 2: Figure S1.** Volcano plot showing significantly differentially expressed proteins in 5 pairs of LSCC tissues and adjacent nontumor tissue. The pink circles on both sides represent 1826 significantly differentially proteins, the absolute fold change is ≥1.2, and the *p*-value is < 0.05.

**Additional file 3: Table S2.** The top 6 mRNAs based on fold change and *p*-value.

**Additional file 4: Figure S2.** IHC was performed to investigate the expression of TMBIM6 in 122 pairs of LSCC samples.

**Additional file 5: Figure S3.** Kaplan-Meier survival analysis suggested that patients with high TMBIM6 expression in LSCC had a worse prognosis.

**Additional file 6: Figure S4.** a The expression efficiency of IGF2BP2 after knocked down the IGF2BP2 in LSCC cells. b The efficiency of IGF2BP2 expression after IGF2BP2 overexpressed in LSCC cells.

**Additional file 7: Figure S5.** a and b qRT-PCR results of TMBIM6 expression after ablation or overexpression of IGF2BP2 in LSCC cells.

**Additional file 8: Figure S6.** a-c The relationship between the expression level of IGF2BP3 and clinicopathological characteristics in LSCC patients.

**Additional file 9: Figure S7.** CCK-8 assays were conducted to examine the cell proliferation ability in LSCC cells after transfection with shCtrl and/or shRBM15 and/or RBM15 vectors.

## Data Availability

The data and materials of this study are available from the corresponding authors for reasonable requests.

## Abbreviations

ALKBH5: AlkB homolog 5, RNA demethylase
ATAD3A: ATPase family AAA domain containing 3A
CASP9: Caspase 9
CCK-8: Cell Counting Kit-8
CPNE5: Copine 5
CRC: Colorectal Carcinoma
DMEM: Dulbecco’s modified Eagle’s medium
FBS: Foetal Bovine Serum
FTO: FTO alpha-ketoglutarate dependent dioxygenase
GAPDH: Glyceraldehyde-3-phosphate dehydrogenase
HMG20A: High mobility group 20A
HNRNP: Heterogeneous nuclear ribonucleoprotein
HNSC: Head and neck squamous cell carcinoma
IGF2BP3: Insulin like growth factor 2 mRNA binding protein 3
IHC: Immunohistochemical
LSCC: Laryngeal squamous cell carcinoma
MAP 3 K6: Mitogen-activated protein kinase kinase kinase 6
MeRIP-seq: Methylated RNA immunoprecipitation sequencing
METTL14: Methyltransferase like 14
METTL16: Methyltransferase like 16
METTL3: Methyltransferase like 3
m6A: N6-methyladenosine
mRNA-seq: mRNA transcriptomic sequencing
NHBEC: Normal human bronchial epithelial cell
OS: Overall Survival
PBS: Phosphate buffered saline
qRT-PCR: Quantitative real-time PCR
RBM15: RNA binding motif protein 15
RBM15B: RNA binding motif protein 15B
RFS: Relapse Free Survival
RIP: RNA immunoprecipitation
SCC: Squamous cell carcinomas
TMBIM6: Transmembrane BAX inhibitor motif containing 6
Wnt: Protein Wnt-2
WTAP: WT1 associated protein
YTHDF1: YTHN6-methyladenosine RNA binding protein 1
