# Supplementary material for: RBM15 facilitates laryngeal squamous cell carcinoma progression by regulating TMBIM6 stability through IGF2BP3 dependent
Source: J Exp Clin Cancer Res. 2021 Feb 26;40:80. doi: 10.1186/s13046-021-01871-4 (PMC7912894; doi:10.1186/s13046-021-01871-4)
Supplement: Supplementary file 1 — Additional file 1: Table S1. The primers used in this study. [file 13046_2021_1871_MOESM1_ESM.pdf]

Table S1. Primers used in the study.

Gene	Sequences	Applications
TMBIM6	F: 5’-TCATATAACCCCGTCAACGC-3’<br>R: 5’-CAAATCCAGCAAGAAGTCCC-3’	MeRIP -qPCR
ATAD3A	F: 5’-TTTCCGTCACCCGTTTGC-3’<br>R: 5’-ATTCAGGGCGTCCTTGGC-3’	MeRIP -qPCR
CASP9	F: 5’-GGTCGCTAATGCTGTTTCGGT-3’<br>R: 5’-GTGCAAGATAAGGCAGGGTGAG-3’	MeRIP -qPCR
MAP3K6	F: 5’-GTGCTTCCCCAGCGTAGG-3’<br>R: 5’-TCCGTGTACTCATAATCAAACTCC-3’	MeRIP -qPCR
CPNE5	F: 5’-CCCCTCCTCAATTTCTTGG-3’<br>R: 5’-GTACCCCTCTTTCACCCGT-3’	MeRIP -qPCR
HMG20A	F: 5’-CCTTCCCCCTCCTTAGTGA-3’<br>R: 5’-TCTGTGAATAGCCACCGCA-3’	MeRIP -qPCR
RBM15	F: 5’-GGCTGCCTGAGGAGAGTGGAG-3’<br>R: 5’-CGGCTACTGCTCAATTCTGGACTG-3’	qRT-PCR
TMBIM6	F: 5’-GCACGGACTCTGGAACCATGAAC-3’<br>R: 5’-GCAGCCGCCACAAACATACAAAG-3’	qRT-PCR
ATAD3A	F: 5’-ATCAATGAGATGGTCCACTTCG-3’<br>R: 5’-CCGGCTTAAGAACATACTTGTC-3’	qRT-PCR
CASP9	F: 5’-GACCAGAGATTCGCAAACCAGAGG-3’<br>R: 5’-AAGAGCACCGACATCACCAAATCC-3’	qRT-PCR

MAP3K6	F: 5’-CTCTACAGCGGCTGAATGAG-3’<br>R: 5’-TGAATCCACATTCAGTTCCTGT-3’	qRT-PCR
CPNE5	F: 5’-AAAGACATGTTTTCCAAGTCCG-3’<br>R: 5’-ACGTCGTATAGATCAAAACGGA-3’	qRT-PCR
HMG20A	F: 5’-GGAGAGTAATGATCTGGCTACC-3’<br>R: 5’-ACAAATTCTGGATTGTTGGTGG-3’	qRT-PCR
IGF2BP3	F: 5’-GAGGCGCTTTCAGGTAAAATAG-3’<br>R: 5’-AATGAGGCGGGATATTTCGTAT-3’	qRT-PCR
GAPDH	F: 5’-TGAACGGGAAGCTCACTGG-3’<br>R: 5’-TCCACCACCCTGTTGCTGTA-3’	qRT-PCR
TMBIM6-2222	F: 5’ TTCAGTGTGAGAGGATCGAGC 3’<br>R: 5’ CTGAGGCCACTGTGTCAAAC 3’	MeRIP -qPCR
CPNE5-1572	F: 5’ CCATGAAGCTGATGGGTTTG 3’<br>R: 5’ ATGGCCTACGGTCCATGATTA 3’	MeRIP -qPCR

Abbreviation: ATAD3A: ATPase family AAA domain containing 3A; CASP9: caspase 9; CPNE5: copine 5; GAPDH, glyceraldehyde-3-phosphate dehydrogenase; HMG20A: high mobility group 20A; IGF2BP3: insulin like growth factor 2 mRNA binding protein 3; MAP3K6: mitogen-activated protein kinase kinase kinase 6; RBM15: RNA binding motif protein 15; TMBIM6: transmembrane BAX inhibitor motif containing 6
