# Supplementary material for: RBM15 facilitates laryngeal squamous cell carcinoma progression by regulating TMBIM6 stability through IGF2BP3 dependent
Source: J Exp Clin Cancer Res. 2021 Feb 26;40:80. doi: 10.1186/s13046-021-01871-4 (PMC7912894; doi:10.1186/s13046-021-01871-4)
Supplement: Supplementary file 2 — Additional file 2: Figure S1. Volcano plot showing significantly differentially expressed proteins in 5 pairs of LSCC tissues and adjacent nontumor tissue. The pink circles on both sides represent 1826 significantly differentially proteins, the absolute fold change is ≥1.2, and the p-value is < 0.05. [file 13046_2021_1871_MOESM2_ESM.pdf]

Figure S1

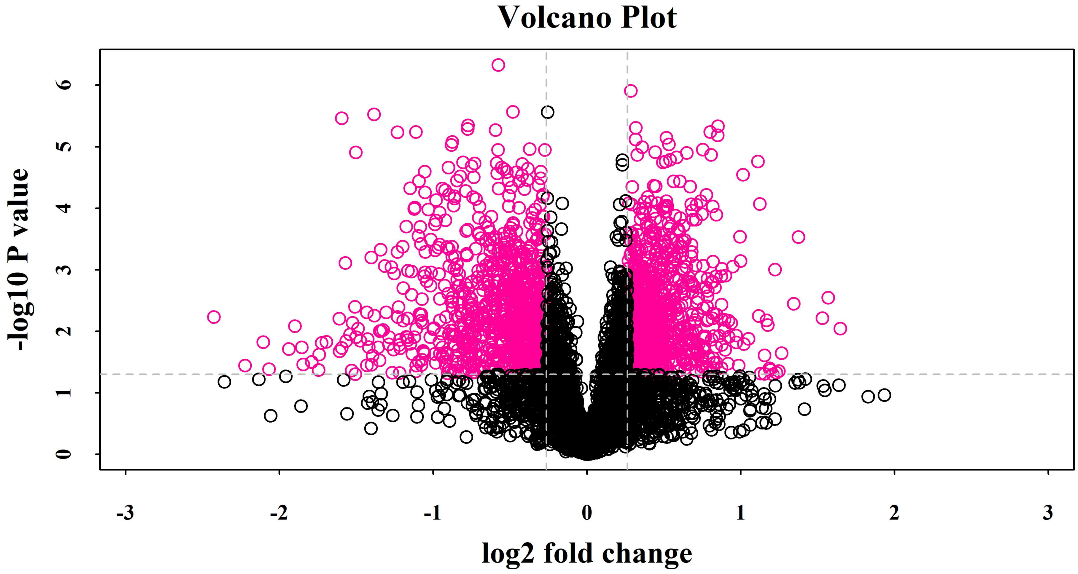

Figure S1. Volcano plot showing significantly differentially expressed proteins in 5 pairs of LSCC tissues and adjacent nontumor tissue. The pink circles on both sides represent 1826 significantly differentially proteins, the absolute fold change is ≥1.2, and the *p*-value is <0.05.
