# Supplementary material for: RBM15 facilitates laryngeal squamous cell carcinoma progression by regulating TMBIM6 stability through IGF2BP3 dependent
Source: J Exp Clin Cancer Res. 2021 Feb 26;40:80. doi: 10.1186/s13046-021-01871-4 (PMC7912894; doi:10.1186/s13046-021-01871-4)
Supplement: Supplementary file 3 — Additional file 3: Table S2. The top 6 mRNAs based on fold change and p-value. [file 13046_2021_1871_MOESM3_ESM.pdf]

Table S2. The top 6 mRNAs with differential m6A methylation modifications were screened out.

Gene	Foldchange(log2-Scaled)	*P*-value(paired t-test)
TMBIM6	0.649519554	0.033421116
ATAD3A	0.626995376	0.049728946
CASP9	0.667971081	0.033006025
MAP3K6	0.617107056	0.000669577
CPNE5	0.614209974	0.020752312
HMG20A	0.736722214	0.029893781

Abbreviation: ATAD3A: ATPase family AAA domain containing 3A; CASP9: caspase 9; CPNE5: copine 5; HMG20A: high mobility group 20A; MAP3K6: mitogen-activated protein kinase kinase kinase 6; TMBIM6: transmembrane BAX inhibitor motif containing 6;
