# Supplementary material for: RBM15 facilitates laryngeal squamous cell carcinoma progression by regulating TMBIM6 stability through IGF2BP3 dependent
Source: J Exp Clin Cancer Res. 2021 Feb 26;40:80. doi: 10.1186/s13046-021-01871-4 (PMC7912894; doi:10.1186/s13046-021-01871-4)
Supplement: Supplementary file 4 — Additional file 4: Figure S2. IHC was performed to investigate the expression of TMBIM6 in 122 pairs of LSCC samples. [file 13046_2021_1871_MOESM4_ESM.pdf]

Figure S2

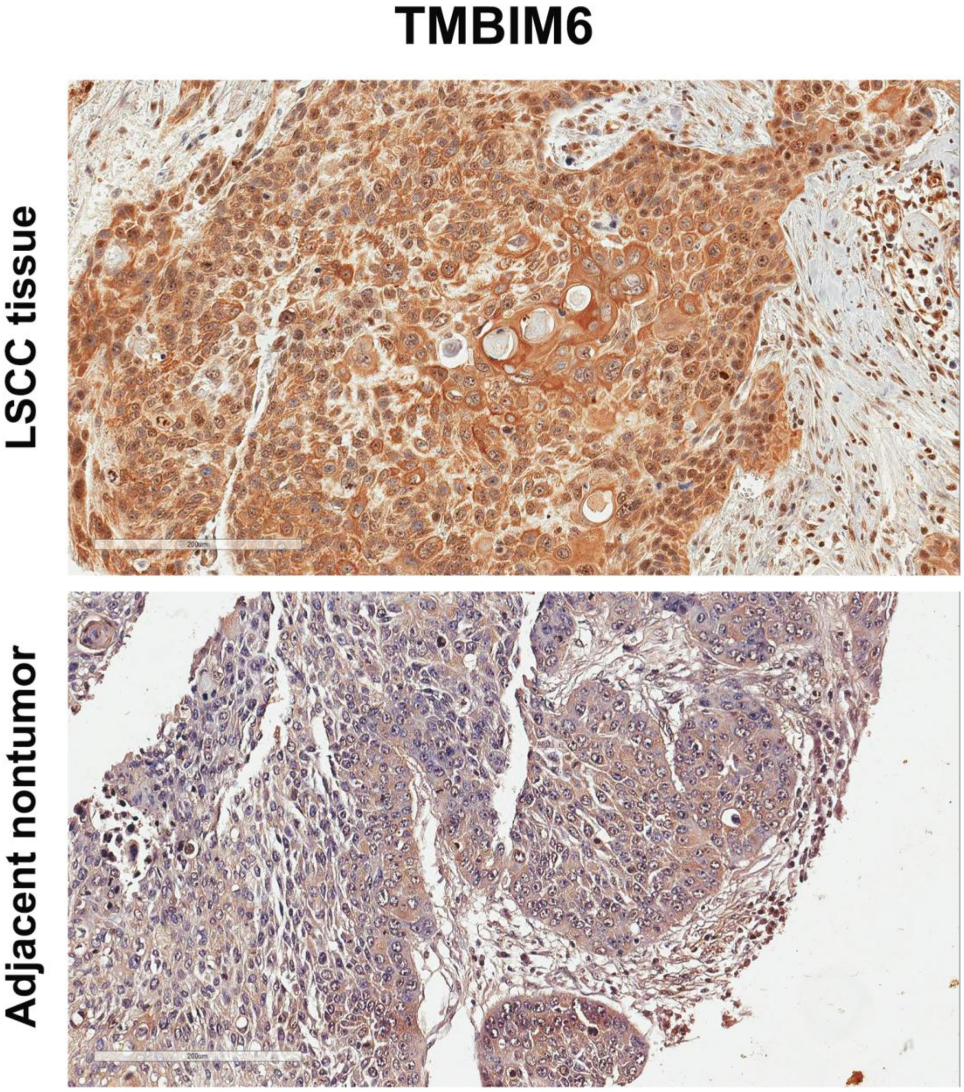

Figure S2. IHC was performed to investigate the expression of TMBIM6 in 122 pairs of LSCC samples.
