# Supplementary material for: RBM15 facilitates laryngeal squamous cell carcinoma progression by regulating TMBIM6 stability through IGF2BP3 dependent
Source: J Exp Clin Cancer Res. 2021 Feb 26;40:80. doi: 10.1186/s13046-021-01871-4 (PMC7912894; doi:10.1186/s13046-021-01871-4)
Supplement: Supplementary file 5 — Additional file 5: Figure S3. Kaplan-Meier survival analysis suggested that patients with high TMBIM6 expression in LSCC had a worse prognosis. [file 13046_2021_1871_MOESM5_ESM.pdf]

**Figure S3**

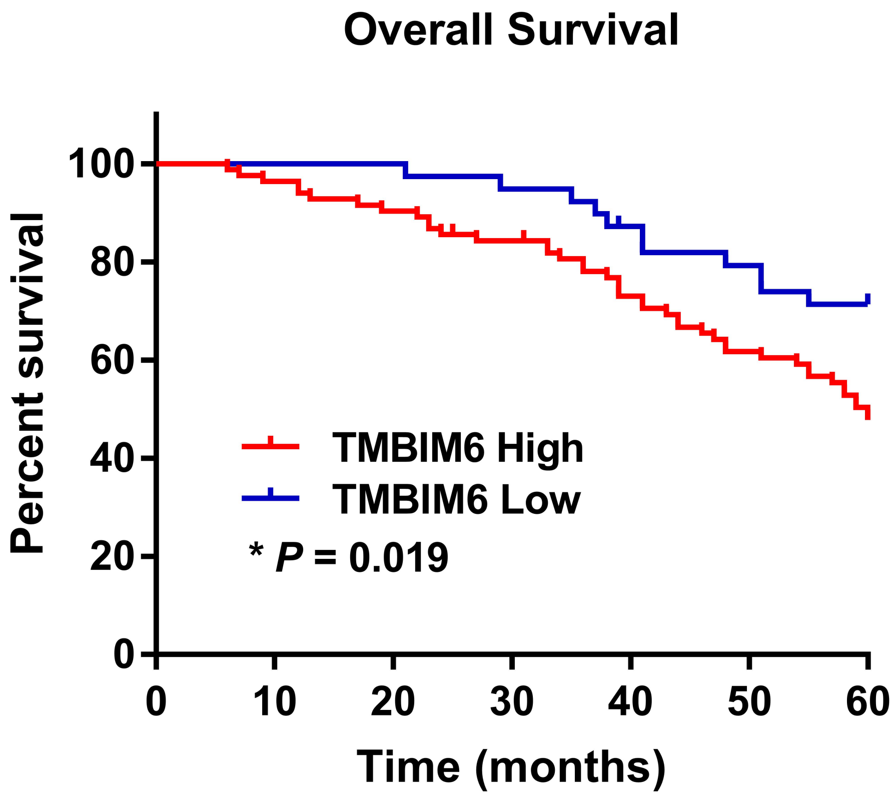


**Figure S3.** Kaplan-Meier survival analysis suggested that patients with high TMBIM6 expression in LSCC had a worse prognosis.
