# Supplementary material for: RBM15 facilitates laryngeal squamous cell carcinoma progression by regulating TMBIM6 stability through IGF2BP3 dependent
Source: J Exp Clin Cancer Res. 2021 Feb 26;40:80. doi: 10.1186/s13046-021-01871-4 (PMC7912894; doi:10.1186/s13046-021-01871-4)
Supplement: Supplementary file 6 — Additional file 6: Figure S4. a The expression efficiency of IGF2BP2 after knocked down the IGF2BP2 in LSCC cells. b The efficiency of IGF2BP2 expression after IGF2BP2 overexpressed in LSCC cells. [file 13046_2021_1871_MOESM6_ESM.pdf]

Figure S4

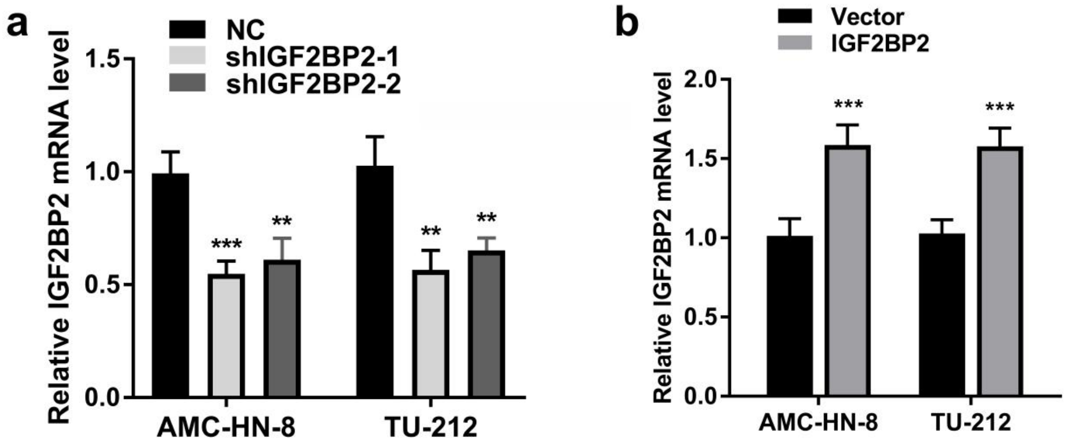

Figure S4. a The expression efficiency of IGF2BP2 after knocked down the IGF2BP2 in LSCC cells. **b** The efficiency of IGF2BP2 expression after IGF2BP2 overexpressed in LSCC cells.
