# Supplementary material for: RBM15 facilitates laryngeal squamous cell carcinoma progression by regulating TMBIM6 stability through IGF2BP3 dependent
Source: J Exp Clin Cancer Res. 2021 Feb 26;40:80. doi: 10.1186/s13046-021-01871-4 (PMC7912894; doi:10.1186/s13046-021-01871-4)
Supplement: Supplementary file 7 — Additional file 7: Figure S5. a and b qRT-PCR results of TMBIM6 expression after ablation or overexpression of IGF2BP2 in LSCC cells. [file 13046_2021_1871_MOESM7_ESM.pdf]

Figure S5

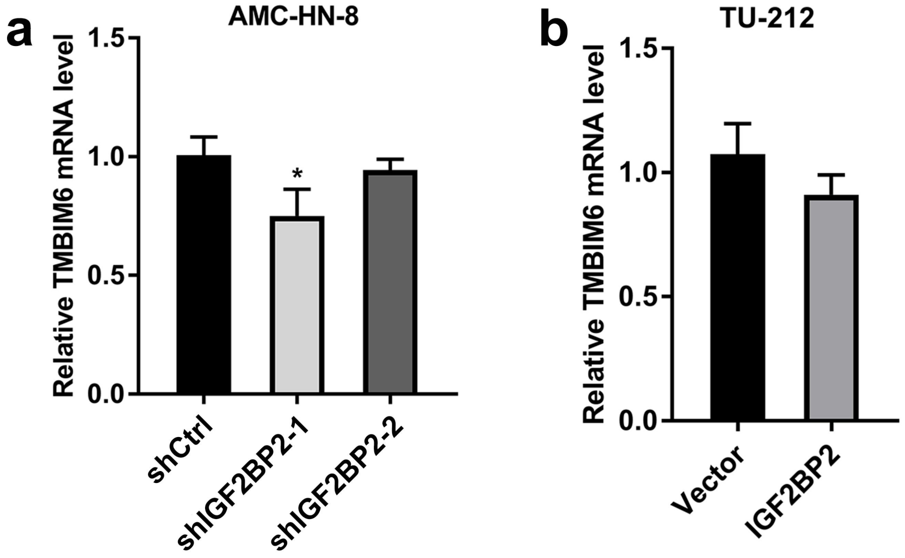

Figure S5. a, b qRT-PCR results of TMBIM6 expression after ablation or overexpression of IGF2BP2 in LSCC cells.
