# Supplementary material for: RBM15 facilitates laryngeal squamous cell carcinoma progression by regulating TMBIM6 stability through IGF2BP3 dependent
Source: J Exp Clin Cancer Res. 2021 Feb 26;40:80. doi: 10.1186/s13046-021-01871-4 (PMC7912894; doi:10.1186/s13046-021-01871-4)
Supplement: Supplementary file 8 — Additional file 8: Figure S6. a-c The relationship between the expression level of IGF2BP3 and clinicopathological characteristics in LSCC patients. [file 13046_2021_1871_MOESM8_ESM.pdf]

Figure S6

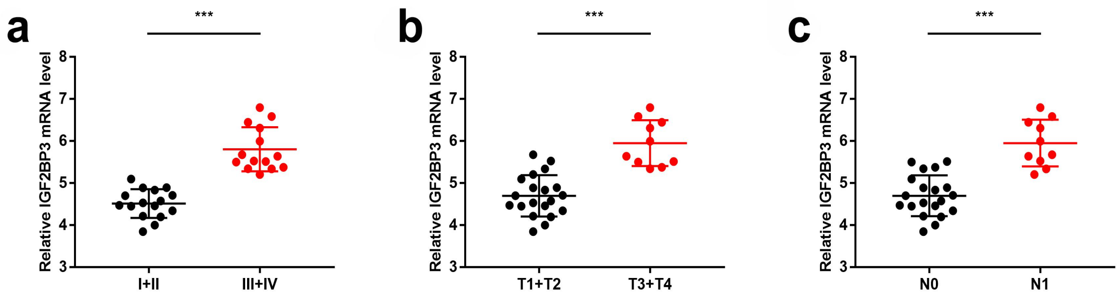

Figure S6. a-c The relationship between the expression level of IGF2BP3 and clinicopathological characteristics in LSCC patients.
