# Supplementary material for: RBM15 facilitates laryngeal squamous cell carcinoma progression by regulating TMBIM6 stability through IGF2BP3 dependent
Source: J Exp Clin Cancer Res. 2021 Feb 26;40:80. doi: 10.1186/s13046-021-01871-4 (PMC7912894; doi:10.1186/s13046-021-01871-4)
Supplement: Supplementary file 9 — Additional file 9: Figure S7. CCK-8 assays were conducted to examine the cell proliferation ability in LSCC cells after transfection with shCtrl and/or shRBM15 and/or RBM15 vectors. [file 13046_2021_1871_MOESM9_ESM.pdf]

Figure S7

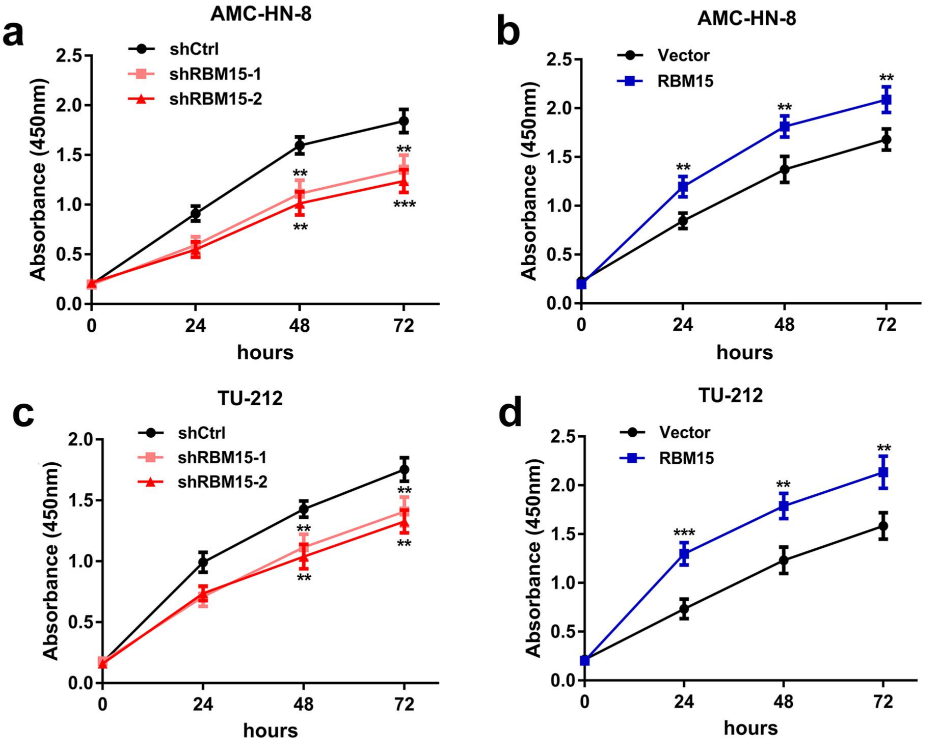

Figure S7. a-d CCK-8 assays were conducted to examine the cell proliferation ability in LSCC cells after transfection with shCtrl and/or shRBM15 and/or RBM15 vectors.
